# Energy poverty in the Energy Community region: Interrogating policy formulation and coverage

**DOI:** 10.1177/09697764231162229

**Published:** 2023-04-25

**Authors:** Stefan Bouzarovski, Jurica Brajković, Slavica Robić, Charlotte Brown, Ivana Vuchkova

**Affiliations:** University of Manchester, UK; Energy Institute Hrvoje Pozar, Croatia; North-West Croatia Regional Energy and Climate Agency, Croatia; ARUP, UK; Friedrich-Ebert-Stiftung Skopje, North Macedonia

**Keywords:** Energy policy, energy poverty, former Soviet Union, Georgia, Montenegro, regional inequality, Serbia, Southeastern Europe, Ukraine, Western Balkans

## Abstract

The capacity of the state to develop and implement policy at the complex nexus of energy infrastructure, social inequality and housing is indicative of the political priorities of governing structures and, by extension, the nature of statecraft more generally. We compare and contrast the energy poverty amelioration policies of two former Yugoslav and two post-Soviet states located outside the European Union, but seeking to join its regulatory sphere – Serbia, Montenegro, Ukraine and Georgia – against the background of deep and persistent patterns of domestic energy hardship. We are particularly interested in uncovering the time horizons, socio-technical systems and target constituencies of different policy measures, as well as energy sector–specific responses to the COVID-19 pandemic. We find that most states in the region have done little to address some of the more substantive challenges around improving housing quality, energy efficiency and gender inequality. However, energy poverty is present in the policy lexicon of all case study countries, and Ukraine, in particular, has advanced a number of more sophisticated approaches and programmes.

## Introduction

Much has been written about the drivers and experience of energy poverty – a condition characterized by the inability to secure needed energy services in the home (Sareen et al., 2020). In Europe, after a long period of limited public recognition and awareness, there is now a committed transnational polity dedicated to the issue (Healy, 2017; Rodriguez-Alvarez et al., 2021). Provisions to detect and address energy poverty have been integrated in a variety of regulatory, policy and legal acts. The establishment of a European Energy Poverty Observatory – and its follow-up, the European Energy Poverty Advisory Hub – has strengthened both the institutional capacities and decision-support systems of relevant governmental agencies (Bouzarovski et al., 2021). In parallel, the numbers and types of stakeholders engaged in the sector are rapidly increasing, with problems of domestic energy vulnerability gaining greater traction and visibility as a result of the COVID-19 pandemic and the Russian invasion of Ukraine (Carfora et al., 2022).

Nevertheless, significant gaps in knowledge, practice and understanding remain. One of these pertains to the lack of practical and theoretical knowledge on energy poverty patterns and policies in the ‘Energy Community’ (EnC) area (formerly known as the ‘Energy Community of South-Eastern Europe’) which encompasses 12 non–European Union (EU) states in the Western Balkan and Black Sea regions, as well as Norway. Alongside the EU, full EnC members (‘Contracting Parties’) include Albania, Bosnia, Georgia, Kosovo, North Macedonia, Moldova, Montenegro, Serbia and Ukraine, while Armenia, Turkey and Norway are ‘observers’. The EnC’s principal purpose is the expansion of the EU’s common energy market to neighbouring countries, via a variety of policy and legal instruments. Its operations in Southeastern Europe (SEE) initially arose out of a desire to implement the EU’s emergent energy security policy in its wider neighbourhood, while assisting Contracting Parties with the post-war reconstruction of their economies (Tangör and Sari, 2022). In practice, the EnC has been primarily focused on promoting regulatory compliance with EU electricity and gas legislation via different forms of ‘rule transfer’ (Padgett, 2011), often through active enforcement and dispute settlement procedures (Verhagen, 2019). In more recent years, the Community’s work – and that of its Contracting Parties – has expanded beyond the steering of energy markets into the promotion of ‘just transitions’ (Bouzarovski, 2022), by addressing renewable energy development, energy efficiency and energy inequalities. This reflects the EU’s expanding energy policy priorities more widely (Skjærseth, 2021).

The formulation and implementation of integrated energy poverty policies is predicated upon complex forms of institutional coordination among multiple sectors and stakeholders. Our decision to explore such measures in the EnC’s Contracting Parties stems from a desire to understand the deeper stories that they tell about the broader practices of energy governance in the region (Vuchkova, 2020), in four respects. First, policy decisions undertaken by the EnC’s Contracting Parties in alleviating energy poverty are relevant in understanding the regulatory structures that underpin infrastructural management, as well as varying institutional capacities to address challenges at the energy–justice interface. Second, energy poverty policies, by virtue of their intersectoral nature, offer deeper insights into the political priorities of governing structures, and by extension the nature of statecraft more generally (Creutzfeldt et al., 2020; Teschner et al., 2020). Third, it is necessary to understand the decision-making trajectories followed by different EnC countries in relation to energy poverty alleviation – an area that has seen limited research to date, even if countries in the region are characterized by much higher rates of domestic energy deprivation than the European average (Bouzarovski et al., 2021; Robić et al., 2017; United Nations Economic and Social Commission for Asia and the Pacific [UNESCAP], 2021). Fourth, there is a need to explore why SEE states are lagging behind in implementing energy restructuring and low-carbon transition policies more generally.

We offer a comparative analysis of energy poverty amelioration policies within the EnC region, in the context of the wider organizational and socio-technical trajectories that underpin energy inequalities (Axon and Morrissey, 2020). Conceptually, the article builds on procedural and recognition justice principles as they relate to wider patterns of household energy use (Feenstra and Özerol, 2021; Simcock et al., 2021a; Stojilovska, 2021). We understand energy justice as a process that ‘fairly distributes both the benefits and burdens of energy services, and one that contributes to more representative and inclusive energy decision-making’ (Sovacool et al., 2017: 677). The principles of inclusion and influence (Simcock, 2016) are central to our analysis: they refer to the question of who is targeted and seen as worthy of recognition in energy policy development and implementation.

Focusing on two former Yugoslav (Serbia and Montenegro) and two post-Soviet (Ukraine and Georgia) states, we uncover the types of policy choices that have been undertaken in different decision-making contexts, and the manner in which these relate to broader typologies of domestic energy deprivation. We base the selection of these four countries on their diverse, yet broadly representative characteristics in relation to the wider EnC space: they provide insights into different patterns and levels of economic and infrastructural development within their respective former post-socialist polities (Serbia vs Montenegro, and Ukraine vs Georgia), are all of different size (from over 40 million people in Ukraine to fewer than 1 million in the case of Montenegro), and are in different stages of the EU accession process (with Montenegro being arguably the most proximate, and Georgia the most distant). Developing the aforementioned energy justice principles in relation to the achievement of equity and ‘fairness in the targeting of solutions’ (Hernández et al., 2022: 134), we examine differences in policy aims and ingredients to discuss why and how particular pathways of energy poverty amelioration have been followed by the case study countries. We also pay attention to energy assistance measures developed during the COVID-19 pandemic, as a reflection of social support priorities. Throughout all of these examinations, our underlying intention is to reveal the broad-level geographical disparities that are arising against the background of the region’s shared post-socialist energy development legacies.

Methodologically, the article is based on a comparative review of relevant policies identified by the EnC through a systematic survey (Ban et al., 2021) that sourced information directly from national governments and official sources. The survey collected information on the relevant policies with the aid of publicly available information, as well as direct contacts with state representatives from the Contracting Parties. We used the data from the survey to identify energy poverty–relevant measures and approaches within each study country, after which the policies were interrogated with the aid of a comparative approach, incorporating thematic interpretive analysis techniques (Peterson, 2017). The policies were examined to understand which mechanisms were being used to address energy poverty, which groups were targeted, and whether they were aimed at providing short-term relief or longer-term reductions in the occurrence of energy poverty. The results of these analyses were grounded in a purposive review of the relevant literature on energy poverty in Eastern and Central Europe (ECE). It should be noted that the research was undertaken prior to the Russian invasion of Ukraine, and thus part of the results presented here may not reflect the rapidly changing situation on the ground.

The article consists of three sections, aside from the introduction. We first examine existing knowledge on the emergence and nature of domestic energy inequities in post-socialist states, with a particular focus on states emerging from the former Yugoslavia and the former Soviet Union. The article then moves onto an overview of energy poverty patterns in the case study countries, using broad-level statistical indicators. The subsequent two sections of the article explore relevant energy poverty policies in relation to (1) temporal horizons and (2) socio-technical targeting. In line with the energy justice principles outlined above, we posit that a given policy’s ability to successfully tackle the root causes of energy poverty increases if its constituent measures (1) extend over a longer period of time, (2) cover a larger group of social groups while encompassing a greater number of dominant energy carriers (a criterion that we term ‘extent of targeting’), and (3) are resourced with a level of funding that can allow for deep energy-efficiency upgrades (in other words, ‘depth of targeting’). The conclusion situates the findings of the policy analysis within the context of broader geographical differences along infrastructural and political lines.

## Domestic energy inequalities in post-socialism: emergent geographies of difference

The research and policy landscape on energy poverty in Europe has been fundamentally transformed over the past 10 years. Compared to the previous period – when energy poverty was interpreted in geographically and conceptually narrow terms – there is now a much more nuanced and detailed understanding of the underpinnings of residential energy inequalities at the local, regional, national and European scales (Bouzarovski et al., 2012; Kyprianou et al., 2019; Primc and Slabe-Erker, 2020). Policy and practice on the issue have moved beyond blunt income support or price subsidy measures onto more comprehensive measures to improve the energy efficiency of the housing stock, the development of targeted support mechanisms for vulnerable groups, and building the capacity of actors who are engaged in the sector (Dobbins et al., 2019; Scarpellini et al., 2017). There has also been extensive scholarship aimed at understanding the connections between energy poverty, on one hand, and the governance of infrastructural development, the housing stock, low-carbon transformations, social welfare and public health (Li et al., 2021; Oliveras et al., 2021), on the other hand.

Existing work on domestic energy inequalities has found significant regional differences across Europe, with Southern European and post-socialist countries generally facing much greater problems regarding household-level poverty and exclusion (Bouzarovski et al., 2021). ECE contains some of Europe’s most vulnerable populations (Robić et al., 2017), particularly in a number of Balkan and, to a lesser extent, Baltic states (Milstead and Miles, 2011). This can be attributed to the broader economic and infrastructural contingencies inherited from the centrally planned economy, as well as the specifics of energy production and consumption in post-socialist countries more broadly. The political and technical legacies of socialism, characterized by under-investment in the housing stock and low residential energy efficiencies, have played a key role. Post-socialist energy reforms have often exacerbated the situation due to the removal of universal price subsidies and the expansion of income poverty, pushing greater numbers of households in energy hardship (Jiglau et al., 2020; Karpinska and Śmiech, 2020; Koďousková and Bořuta, 2022; Simcock et al., 2021a).

Most ECE countries that are inside the EU have sought to overcome the path-dependencies of the socialist political economy by addressing both the drivers and consequences of energy poverty. International regulatory and financial support has played a key role here, as has the EU accession process more specifically. A number of states in Central Europe have advanced ambitious housing renovation programmes, while improving the depth and targeting of social support mechanisms (Földváry et al., 2017; Hrovatin and Zorić, 2018; Turecki et al., 2022). Elsewhere in the region – and particularly in non-EU post-socialist states – energy poverty remains pervasive, and has been further worsened by trends unfolding during the post-socialist transformation: political instability and military conflict, declining real incomes, increasing social inequalities, and slow progress on the upgrading of the housing stock and energy systems (Maxim et al., 2017; Stojilovska et al., 2021).

Within ECE, but also the Global North more broadly, the demographics of people who are energy poor are broader than those with low incomes – mainly because energy poverty is not just caused by low incomes but is also dependent on energy inefficient housing, energy needs and energy supply (Middlemiss, 2022). However, because energy poverty has so many causes and effects, it is difficult to address it via a single measure. It is especially hard to capture households with complex underlying vulnerabilities around mental and physical health, gender, ethnicity, disability, age, disconnections from the energy system and location (Bouzarovski et al., 2022; Simcock et al., 2021b; Snell et al., 2015). In the immediate term, households may need financial support or help with purchasing fuel, but this is not a sustainable approach – especially if fuel use is polluting. Residential energy-efficiency improvements are most effective in addressing energy poverty in the medium to long term, as are changes in heating and cooling systems (Energy Sector Management Assistance Programme, 1999; Thomson et al., 2019).

The transformation of heating systems is one of the greatest challenges faced by Western Balkan and Black Sea countries alike. Pavlović et al. (2021) find that about 25 per cent of households in Serbia are supplied from district heating systems, while the rest (75%) rely on individual heating, primarily based on solid fuels (biomass and coal). Their research points to the ‘obsolescence of household heating systems, low efficiency, insufficient investment in thermal insulation in the past, and lack of financial resources for investing in sustainable heating’ (p. 1). Montenegro, while facing similar problems, has generally seen a small decrease in energy poverty rates, possibly as a result of changing energy production and price patterns (Streimikiene, 2022). In Ukraine, where the achievement of adequate levels of domestic heating is a key challenge, the pandemic has led to rapidly increasing utility bill arrears while compounding a set of difficult circumstances associated with high levels of income poverty and fossil gas prices (Goncharuk et al., 2021; Petrova et al., 2013). Energy poverty in Georgia is pervasive and has a strong rural dimension, linked to the lack of adequate infrastructure service provision – unlike other former Soviet states, Georgia dismantled its district heating infrastructure during the 1990s (UNESCAP, 2021).

Within the Western Balkans, there are also differences among and within countries. Rural areas are particularly vulnerable as people living in them inhabit low-quality housing and are often forced to rely on polluting fossil fuels (such as fuelwood and coal) for heating (Petrova and Prodromidou, 2019; Stojilovska et al., 2021). In urban areas, residents of large housing estates equipped with district heating may often find it expensive to purchase heat and hot water. Informal settlements at the urban fringe, present in many Southeastern European cities, are among the most vulnerable as the people who live in them are vulnerable and discriminated along multiple axes: housing, income, ethnicity and social participation.

Across the post-socialist space, there is a growing recognition that energy-related inequalities intersect with wider income, housing factors and socio-demographic circumstances to produce complex and extensive forms of deprivation that often remain below the radar of policy and research. One of the entrenched challenges encountered by practitioners and analysts working in this area has been the lack of adequate statistics and indicators to identify and monitor the gender–energy poverty relationship; because most statistical data are only provided at the household scale, it is difficult to detect gendered aspects of intra-household vulnerability, some of which may be more private or personal (Petrova and Simcock, 2019; Robinson, 2019).

Policy efforts to mainstream gender issues in energy poverty debates have been aided by the growing prominence of these issues in the agendas of European institutions (Clancy et al., 2003, 2017). Nevertheless, data coverage and overall knowledge of the question remains poor. In Western Balkan and Black Sea countries, energy poverty problems and gender inequalities are strongly rooted in historic socio-cultural and economic circumstances (Bîrsănuc, 2022). Specificities and challenges at the gender–energy poverty nexus in the region include the relatively high labour participation of women – despite continued domestic inequalities – as well as poor social safety nets provided by the state, in addition to continued forms of discrimination in all aspects of the energy sector, and particularly towards ethnic minority women. A rare policy brief exploring links between air pollution and energy poverty in Bosnia (Strambo et al., 2021) connects these problems with gender inequalities, arguing that the gendered division of labour in the country (due to which women tend to undertake a larger share of reproductive tasks in the household) also means that women are more likely to suffer adverse exposure to indoor air pollution. Measures to cope with air pollution also have adverse impacts on women’s working hours and labour opportunities.

To summarize, despite the substantive body of research on the determinants of energy poverty in post-socialist states, context-specific knowledge on the political-economic and lived dimensions of the problem beyond the EU borders is limited. Even less is known about the regulatory and policy aspects of the issue as they relate to wider trajectories of decision-making by the state.

## Energy poverty patterns in the case study countries

Understanding the extent and depth of energy poverty in the four states reviewed in this article requires considering relevant measures of domestic energy deprivation and energy inequality available at the national scale. The most frequent measures used in this context are (1) the energy burden, which measures the share of household expenses as a share of total household incomes; (2) the ‘2M’ indicator, which quantifies number of households whose absolute energy expenditure is below the national median of energy expenditures, thus measuring the ‘underconsumption’ of energy services; and (3) the ‘M/2’ indicator, which establishes how many households have an energy burden that is twice higher than the national median energy burden. National statistical agencies in the case study countries gather expenditure data via Household Budget Surveys (HBS) or the Survey of Living Conditions of Households (SLCH) in Ukraine; combined with other demographic and economic data, this allows for identifying the groups that suffer from disproportionately high or low energy costs. The 2M indicator is judged to provide a more complete picture of energy poverty in Western Balkan and Black Sea countries, where income inequality levels are high.

Another energy poverty method involves establishing if people experience some of the objective symptoms of energy poverty, such as being unable to pay their energy bills on time; or living in a house with mouldy walls, condensation or a leaking roof. Developing this further, respondents can be asked about their subjective (also known as ‘consensual’) impressions of the level and quality of energy service reached in the home (e.g. if they can keep the home adequately warm), as reported by national statistical agencies, in addition to Eurostat’s Statistics on Income and Living Conditions (SILC) survey (Rademaekers et al., 2016; Schuessler, 2014; Thomson et al., 2017; Tirado Herrero, 2017).

The extent to which these indicators are suitable in the case study context depends on their wider parameters described above, as well the specific circumstances and constraints present in the region (Table 1). We established that standardized data were available for only 1 year – 2019 – across the four case study countries, with the energy burden indicator being the only comparable measure. However, it was possible to calculate the remaining indicators – M/2, 2M, the share of households with arrears on utility bills, the share of households living in poor-quality homes (with evidence of damp, mould and condensation), the share of households reporting an inability to keep adequately warm – for three of the case study countries (Table 2).

**Table 1.** Availability of data for different energy poverty measures across the case study countries.

Country	Expenses based			Objective		Subjective	Source
	Energy burden	M/2	2M	Bill arrears	Poor housing	Adequate warmth	
Ukraine	Y	Y	Y	Y	Y	Y	SLCH
Montenegro	Y	N	N	Y	Y	Y	HBS, SILC
Serbia	Y	Y	Y	Y	Y	Y	HBS, SILC
Georgia	Y	Y	Y	N	N	N	HBS

SLCH: Survey of Living Conditions of Households; HBS: Household Budget Surveys; SILC: Statistics on Income and Living Conditions.

**Table 2.** Energy poverty indicators across the four case study countries.

Totals	Georgia (%)	Ukraine (%)	Serbia (%)	Montenegro (%)
2M	23	12	12	NA
M/2	19	14	14	NA
Share of households with arrears on utility bills	NA	20	12	31
Poor-quality homes	NA	9	19	27
Unable to keep adequately warm	NA	23	11	11
Energy burden^a^	17	8	12	13
Share of households satisfying any 2 criteria	NA	15	12	15
Share of households satisfying all 3 criteria	NA	4	3	3

Source: SLCH, SILC, HBS data.

SLCH: Survey of Living Conditions of Households; HBS: Household Budget Surveys; SILC: Statistics on Income and Living Conditions.

a Data from Montenegro and Ukraine refer to the weighted and equivalized mean share of energy expenditure in household disposable income. Data from Georgia refer to the equivalized and weighted mean share of energy expenditure in income. Data from Serbia refer to the median share of energy expenditure in income.

Overall, we found that the 2M and M/2 indicators were particularly high in Georgia, pointing to the large share of households living with low incomes. The upper bound value of the share of energy-poor households in this country reached one quarter of the sample, translating into an estimated number of 274,000 households. In Ukraine, the share of energy-poor households ranged between 8 and 23 per cent of all households, suggesting that there could be up to 3.4 million energy-poor households in the country. In Montenegro, poor-quality homes account for over one quarter of the inhabited housing stock, with nearly one-third of households reporting late payments of energy bills. This indicates that up to 62,000 households could be energy poor. Equivalent values were somewhat lower in Serbia, even if 19 per cent of households – living in approximately 456,000 homes – found themselves in residential environments conducive to energy poverty. However, it was difficult to establish a common trend across the data. While energy burdens were particularly high in Georgia, a record number of households in Ukraine reported living in cold homes. In the Western Balkans, housing quality was reportedly poorer than in the two post-Soviet republics in the study countries, while noting that households in the former, particularly in Montenegro, tend to inhabit single-family homes to a higher extent.

## Measures to combat energy poverty: timescales and types of support

We identified a total of 18 relevant measures across the four study countries (Table 3). When comparing the types of policies available, we detected clear commonalities in the popularity of certain mechanisms for the protection of vulnerable groups. Across the four countries, short-term financial relief measures were dominant. We found a total of 13 policies focused on protecting vulnerable consumers by either providing additional income or by reducing energy expenditure via reductions in final bills, or allocating reduced tariffs to eligible households. Three of these measures can be seen as short-term stop-gap efforts, as they were created or implemented as a response to the special circumstances brought about by the COVID-19 pandemic. Forced lockdowns, working from home and quarantines all impacted household finances, with many individuals losing their jobs. Energy consumption was also affected, due to increasing energy demand as people spent more time at home. In response to this, both Ukraine and Georgia implemented additional measures to help relieve any further strain on vulnerable households.

**Table 3.** A summary of surveyed policies.

Number and name	Country	No. of households	Annual expenditure (mil. EUR)	Carrier type	Time-scale of policy	Eligibility criteria
1. Socially vulnerable families in Georgia (excluding Tbilisi Municipality)	Georgia	65,907	1	Electricity cost subsidy	Short term	Household below certain social score
2. Socially vulnerable families in Tbilisi Municipality	Georgia	45,000	2.3	Subsidy for electricity, water, waste	Short term	Household below certain social score
3. High mountainous settlements	Georgia	80,256	3.6	Electricity cost subsidy	Short term	Households living in upland regions
4. Families with four or more children	Georgia	270	1.1	Non-targeted	Short term	Household below certain social score
5. Mountainous Settlements in Kazbegi and Dusheti Municipality	Georgia	3600	2.8	Natural gas subsidy	Short term	Mountainous Settlements in Kazbegi and Dusheti Municipality
6. Residents living in the villages near occupation borderline	Georgia	13,000	0.99	Heating allowance	Short term	Villages bordering occupied areas
7. Approval of the rules and conditions for subsidizing utility bills	Georgia	NA	NA	Gas, electricity.	Short term (COVID)	Households that consume a maximum of 200 kWh of electricity per month and a maximum of 200 cubic metres of natural gas per reporting month
8. Subsidy for electricity bill	Montenegro	400–700	N/A	Electricity	Short term	Vulnerable consumers based on the social and health status of household members
9. Subsidy for electricity bill	Montenegro	17,000–21,000	2.7	Electricity	Short term	Recipients of material social welfare support and users of social housing, war veterans, disabled individuals
10. Reduction of monthly electricity bill obligation	Serbia	74,615	9,761,574	Electricity	Short term	Income census
11. Reduction of monthly gas bill obligation	Serbia	50	514	Gas	Short term	Income census
12. Reduction of the obligation to pay for utilities in Novi Sad	Serbia	Unknown	Unknown	District heating	Short term	Families three or more children until completing regular education, and expiring for those 26 years of age and older
13. Subsidy for utility products and services in Belgrade	Serbia	Unknown	Unknown	District heating	Long term	Category 1: pensioners with the lowest tier of pensions, households meeting income criteria; Category 2: war veterans including those with war disabilities, and families of the deceased; Category 3: beneficiaries receiving ensured material support and home care meals, households with severe disability and severely ill members
14. Housing subsidies	Ukraine	2.4 mil.	Unknown	All	Short term	Low income
15. Subsidies during the COVID-19 crisis	Ukraine	Unknown	1.4 bln UAH	All	Short term	Recipients of household subsidy, unemployed
16. Subsidies during the COVID-19 crisis – deductions for electricity payments	Ukraine	Unknown	Unknown	Electricity	Short term	Unknown
17. Programme ‘ENERGODIM’ (Energy Efficiency Fund)	Ukraine	68,500	Unknown	All	Long term	Unknown
18. Programme ‘WARM LOAN’	Ukraine	893–853,000	Unknown	Energy-efficiency measures	Long term	Multi-apartment buildings

In Ukraine, two policies were created specifically during, and due to, the COVID-19 pandemic. The first policy, referred to as ‘subsidies during the COVID-19 crisis’, was particularly wide-ranging in terms of scope and mechanisms. This policy included both the extension and fortification of existing support. It prohibited vulnerable customers from being cut off from energy supplies due to bill arrears during the lockdown. The policy also included a 50 per cent increase in ‘social norms’ for the use of certain utilities – centralized water supply and sewerage, hot water supply, gas supply for cooking and water heating, electricity supply – lasting for the months of the quarantine and starting from March 2020. The norms were used to assess subsidy thresholds, whose increases allowed for increased consumption caused by the quarantine. Under the policy, a rise in the basic payment rate for housing and communal services by recipients of housing subsidies was postponed. The second policy created by Ukraine in response to the pandemic focussed on deductions for electricity payments. These deductions were targeted at specific groups such as those reliant on electricity for heating units or not connected to natural gas.

Similarly, Georgia responded to the pandemic with additional policies to protect vulnerable consumers, though their sole pandemic policy was not as comprehensive as those in Ukraine. In Georgia, subsidies were provided for some vulnerable consumers, notably those consuming smaller amounts of energy. This was specifically aimed at households that consume a maximum of 200 kWh of electricity per month and a maximum of 200 cubic metres of natural gas per month. The policy was implemented between November 2020 and February 2021.

We also found several examples of shorter-term measures which were of a more permanent nature and not created in response to the COVID-19 pandemic. These again provided financial aid to vulnerable consumers, and generally focussed on reducing household outgoings for energy costs by reducing bills or providing additional income. Thus, a number of policies aimed to alleviate energy poverty by reducing the total costs of energy for vulnerable consumers, generally by relying on direct bill interventions. In Georgia, bill reductions were achieved in two ways. First, by reducing the tariff that a vulnerable consumer pays for the energy they use. This measure – available primarily to residents of mountainous regions – provided a 50 per cent discount on electricity tariffs for the consumption of up to 200 kWh. A second measure targeted gas expenditure, and also reduced energy outgoings by decreasing the cost of gas. However, instead of a tariff reduction, this policy actually provided a specific amount of free gas to vulnerable consumers.

In Serbia, bill deductions were also used as a method to protect vulnerable consumers. We identified four policies aimed at reducing energy bills. Two national policies were each aimed at decreasing bills for gas and electricity, respectively. For both policies, the size of the bill reduction related to both the total household income and the size of the household. We were also able to locate two local policies. In Novi Sad, families with three or more children received deductions on utility bills – gas, electricity and district heating. The total deduction amount varied depending on the household size and ranged from 30 to 50 per cent. In addition, Serbia developed a subsidy for utility products and services used by vulnerable consumers who are residents of Belgrade.

Other policies provided additional income to households. In Georgia, three policies used this mechanism to direct additional payments to groups that were deemed vulnerable. A policy named ‘Socially vulnerable families in Georgia’ provided a subsidy of €0.015 per kWh for those who resided outside of Tbilisi Municipality and were considered vulnerable. At the same time, vulnerable consumers living within Tbilisi Municipality were covered by a separate subsidy scheme with two different rates. Those deemed more vulnerable in socio-demographic terms were paid more – with subsidies ranging from €3.13 to €6.25 a month – while an additional €6.56 in subsidies were also available to families with four or more children (the total amount received increased per additional child). Finally, residents living in villages near the borders of zones occupied by Russia received a subsidy equating to around $70 from the Georgian Government in winter, in the form of a heating allowance.

The two policies that we identified in Montenegro tackled energy poverty by providing cash payments to vulnerable consumers. One policy was targeted at those households who were not necessarily considered income poor but received social support relating to disability, or the need for domestic care. In 2019, between 400 and 700 households were entitled to this subsidy, which amounted to a 50 per cent deduction in their monthly electricity bill for up to 600 kWh of consumed electricity. A second subsidy was more explicitly income-based, by being targeted at existing beneficiaries of social welfare support, social housing residents and beneficiaries of social welfare support for war veterans. This subsidy provided 40 per cent of the final bill up to a total of €60, and for bills higher than €60, a subsidy equivalent to €24 was foreseen. Due to the design of the policies, beneficiaries of disability support ended up receiving a lower subsidy, which covered 30 per cent of bills up to €60, and €18 on bills above €60.

Ukraine’s only non-COVID-19 energy poverty–related social measure offered additional housing subsidies to low-income families in the form of direct cash payments, so as to cover the cost of housing and utility services. Across the four case study countries, these short-term financial interventions helped relieve some of immediate and more direct financial pressures faced by energy-poor households. However, a number of governments also implemented disconnection bans that would prohibit energy supplies from being suspended due to bill arrears. In Montenegro, the Energy Act uses this mechanism by preventing the interruption of electricity services to vulnerable consumers who require health and social support, while for vulnerable consumers requiring social support, the suspension is in place from the beginning of October until the end of April, regardless of possible overdue bills for consumed electricity. As mentioned above, a disconnection ban was also used in Ukraine as a temporary COVID-19 measure. In Serbia, Georgia and outside of the pandemic within the Ukraine, policies prohibiting the disconnection of energy supplies were not found.

The implementation intervals for each of these measures also varied. We found that statutes that focused on cash payments or bill reductions were only implemented during the winter period – particularly in the case of gas and heating. Georgia’s policy to reimburse the costs of gas supplied to households in highland villages was only active between October and May. Serbia’s deductions in monthly gas bills were only provided between October and March. The time-limited nature of these policies means that they do not possess the capacity to address either the driving forces or the recurrence of energy poverty, even if they do provide some much-needed short-term assistance to vulnerable households.

Only two measures – both developed by the Ukrainian government – were focussed on confronting the structural aspects of energy poverty. Both addressed energy efficiency by providing funds or partial reimbursements for domestic energy improvements. Ukraine’s Energy Efficiency Fund Act promoted the implementation of incentives and assistance measures for improving the energy efficiency of multi-apartment buildings. The Fund was limited in scope, however, and did not provide grants or other types of support for renovating individual houses or multi-apartment buildings where a homeowners’ association has not been established. In addition, we found that a ‘warmth loans’ energy-efficiency programme provided partial compensation for energy-efficiency measures undertaken in individual houses or multi-apartment buildings where a homeowner’s association or a housing construction cooperative has been established. By 2020, the programme stipulated that if shared blocks of flats included families receiving the subsidy, such buildings would receive a weighted average reimbursement of between 40 and 70 per cent for energy-efficiency measures, depending on the number of subsidized apartments. Around 854,000 households participated in the implementation of the programme.

Despite the importance of climate mitigation measures in both reducing carbon emissions and alleviating energy poverty on a longer-term basis, we did not find any further evidence of specific measures to decrease energy expenditures via methods such as support for renewables, energy efficiency or improvements to heating systems.

## Defining the (un)deserving poor: differences in eligibility, cost and types of energy carriers

All the measures that we reviewed were underpinned by qualifying criteria that identified specific target groups and constituencies. We found large differences in the numbers and types of people that were deemed eligible across the four countries, as well as the ways in which suitability for the measures was assessed. Here, we review and discuss the policies in relation to five targeting categories: (1) low income, (2) household size, (3) the presence of small children, (4) social vulnerability and benefit eligibility, and (5) specific energy carriers. These categories reflect our initial analytical framing – if a policy is more ambitious in its socio-demographic coverage and targeting horizons, it is more likely to address the structural causes of disadvantage.

Notably, three of the policies were associated with qualifying criteria based on household income. In 2019, 3.1 million households were eligible for energy-related subsidies in Ukraine, based on being classed as having a ‘low income’. In Serbia, income thresholds were adjusted based on household size, accounting for larger families. Under the ‘Deductions for Monthly Gas Bills’ policy (part of the ‘Ordinance on Energy Vulnerable Consumers’), a one-person household earning less than €126 per month could receive support from the state, rising to €304 for households with six or more members. As a whole, this meant that the costs of the various financial support measures varied significantly. In 2019, the cost of the Serbian policy deduction for monthly electricity bills amounted to a total of €9,761,574.

Rather than just basing eligibility on income and household size, some measures also took numbers of children into account. For example, deductions to utility fees in Serbia’s Novi Sad municipality were available to families with three or more children who are either younger than 26 or still in full-time education. Similarly, in Georgia, additional funding was available to vulnerable families who had four or more children. Several of the policies used pre-existing categorizations or ‘vulnerability ratings’ from wider assessments within the social support system. For example, three measures in Georgia were based on more complex criteria, where household eligibility was judged using a social ‘vulnerability rating’ score. These quantifications were assigned to households that wished to receive social assistance and were designated by an agency representative based on information provided by the applicants. The score was calculated using a wide range of factors relating to the living conditions of households, including the appliances, goods and property owned by the household, as well as energy, gas and water consumption levels in the previous 12 months. The score also took into account the condition of the dwelling, its location (different grades were given depending on whether the household lived in the capital city, a large city, a small city or a rural settlement), the number and age of household members, and health issues faced by household members. All relevant factors were assigned scores and weights, which were then transposed into rated scores using formulas. The scores were then used to determine the extent and amount of support that would be provided by the government.

In a similar manner, Montenegro’s assistance measures were made available to citizens who were already in receipt of other benefits – income support, a domestic care allowance, or a disability allowance. This exemplifies the use of more generic and well-established social assistance criteria to provide energy poverty assistance. In Georgia, some of the financial measures targeted specific groups regardless of income, as exemplified by the inclusion of mountain residents, as well as people living in villages near occupied territories. Consumers living in mountainous settlements only were eligible to receive 700 cubic metres of free gas per month and could receive the 50 per cent tariff discount for a maximum electricity consumption of 200 kWh. In Ukraine, specific COVID-19 benefits were offered to those who received a housing subsidy in the 2019–2020 heating period and to individuals who had lost their jobs. Additional measures in response to COVID-19 to further subsidize electricity, natural gas, drinking water and sewage were available for low-consuming households in Georgia. Specifically, this applied to households that consumed a maximum of 200 kWh of electricity per month and a maximum of 200 cubic metres of natural gas per reporting month. Longer-term energy-efficiency measures were also associated with specific eligibility criteria, involving energy-efficiency standards, the existence of a management structure for residential buildings, or the building typology itself (e.g. the ‘Warmth loans’ programme was available only to the inhabitants of individual family homes).

Financial measures aimed at reducing energy poverty also targeted different types of energy supply. Some approaches involved a specific type of energy source – either gas or electricity. Serbia and Georgia focused specifically on gas, with the aforementioned allowance being targeted at gas use among mountainous settlements. In contrast, some of the measures specifically focused on reductions in the cost of electricity. This type of assistance was found in Georgia, Montenegro and Serbia. We also established that some measures did not focus on a single energy type but rather chose to encompass wider expenditure on housing and utilities, which also includes energy consumption. Such an approach was taken by the two measures in Serbia, where subsidies for utility products and services in Belgrade and deductions on utility fees in Novi Sad covered electricity, gas and district heating. Ukraine’s only financial measure was targeted at multiple utility services – the consumption of electricity, cold and hot water, sewage, as well as gas for cooking and heating purposes. Even though most forms of assistance in Ukraine were financed and provided nationally, local authorities played an important role in disbursing and governing the support. We also found two policies in Serbia and one in Georgia where local authorities took centre stage in governing the programmes. In the case of the latter, electricity subsidies were different for people living in Tbilisi compared to the rest of the country, reflecting the elevated cost of living in the nations’ capital.

## Conclusion

EnC Contracting Parties are increasingly implementing dedicated strategic approaches at the nexus of energy infrastructure, prices and household budgets, against the background of an almost complete absence of such support in the early days of the post-socialist transformation. The policy measures reviewed in this article indicate that the four case study countries in the Western Balkans and Black Sea region have principally developed energy poverty alleviation initiatives by relying on the relatively robust and extensive social assistance systems inherited from the socialist past. In terms of the time horizons of the policies surveyed in this article – the first of our two analytical criteria – short-term stop-gap measures predominate, reflecting the lack of governing capacity to institute more ambitious and far-reaching programmes. As for socio-technical coverage – the second analytical criterion – the surveyed measures have tended to add different forms of income support for households struggling with energy bills to the existing social welfare system. These schemes principally operate at the national scale and involve the targeting of households who have low earnings, or are vulnerable by virtue of increased energy needs (due to larger family sizes as well as the presence of children or disabilities), living in deprived areas (primarily mountainous or politically unstable regions, but also some urban centres – potentially for political reasons) or inhabiting poor-quality housing.

We did, however, detect a number of measures that depart from the usual playbook of treating energy poverty as an issue of income deprivation. Ukraine is a clear leader in this respect, with its development of energy-efficiency programmes that stimulate collective solutions to housing management, the formulation of energy-efficiency standards, and an increased role for local authorities. Georgia’s development of sophisticated vulnerability scores and clear geographical targeting also introduces a level of policy innovation in this regard, even if the relatively untransparent technocratic calculus that underpins them could potentially open the path for marginalization of certain constituencies or various forms of elite capture. At the other end of the scale lie the former Yugoslav republics of Serbia and Montenegro, both of which have been relatively less affected by military conflict, and are formally part of the EU accession process. Surprisingly, the policy arsenal formulated by these two states is relatively conventional and limited, despite the fact that their policy transformations have been overseen and supported by the EU financial and regulatory structures much more explicitly and resolutely compared to states in the post-Soviet space.

As a whole, and comparing the four countries in relation to the key elements of our analysis (by distinguishing between the extent and depth of targeting, and also taking into account COVID-19 amelioration measures), it is clear that Montenegro is a clear laggard among the four case study countries while pre-invasion Ukraine is a leader (Table 4). Indirectly, this suggests that Ukraine may be well placed, in institutional terms, to undertake the post-war reconstruction of its energy sector once the invasion is over. At the other end of the scale, the EU accession process has yet to deliver comprehensive energy poverty policy reforms in Montenegro (and to a lesser extent, Serbia).

**Table 4.** Summative comparison of energy poverty policies in the four case study countries.

Criterion	Georgia	Montenegro	Serbia	Ukraine
Time horizons of policies	Short-term measures predominate	Short-term measures predominate	Short-term measures predominate with some long-term measures	Combination of short- and long-term measures
Extent of targeting	Primarily based on social vulnerability criteria; however, area-based policies also present	Primarily based on social vulnerability criteria	Primarily based on social vulnerability criteria with some geographical targeting	Wide range of criteria used
Depth of targeting	Relatively limited extent of policies	Relatively limited coverage	Reasonably strong coverage	Extensive targeting
COVID-19 support	Short-term support available	No measures identified	No measures identified	Short-term support available

Significant gaps in both policy and understanding remain when it comes to addressing the complex institutional, spatial and economic relations that underpin energy transformations in the EU’s southeastern neighbourhood. The lack of a clear and robust housing policy to address energy poverty stands out across the four case study countries, and particularly in Serbia and Montenegro where the share of poor-quality residential housing is disproportionately high. Another challenge relates to the policy prioritization of housing energy–efficiency upgrades and domestic heat decarbonization within broader state investment approaches – including infrastructure development – as opposed to expanding transfers to income-poor households. The strength of COVID-19 responses, where Ukraine once again is at the highest end of the evaluation scale (unlike Serbia and Montenegro), might prove instructive in this regard. Gender-sensitive measures are equally lacking across the region, even if Georgia’s vulnerability scoring is potentially opening the path for an approach that is more sensitive to intra-household differences.

More broadly, our analysis uncovers additional elements relevant to the process of approaching and identifying the drivers of energy poverty and injustice. First, it draws attention to the need for understanding energy poverty not solely in terms of its immediate underpinnings (particularly energy efficiency and poor-quality housing), but also in relation to the broader governance capacities that lead to particular ‘strategic selectivities’ (Jones, 1997) of the state. Second, it highlights the need for developing more sensitive targeting mechanisms to widen, sharpen and deepen the support available to vulnerable households. Third, our analysis indicates the significant challenges for the development of ambitious, structurally oriented energy poverty alleviation policies across the EnC space, with only Ukraine having made some inroads in this regard. In particular, there is limited scientific knowledge and best practice on the governance structures and intersectoral dynamics associated with the introduction of a more ambitious housing transformation agenda across SEE and the EnC space more widely.
